# Special Issue “Advances in Artificial and Biological Membranes: Mechanisms of Ionic Sensitivity, Ion-Sensor Designs, and Applications for Ion Measurement”

**DOI:** 10.3390/membranes10120427

**Published:** 2020-12-15

**Authors:** Andrzej Lewenstam, Krzysztof Dołowy

**Affiliations:** 1 Faculty of Materials Science and Ceramics, AGH University of Science and Technology, 30-059 Krakow, Poland; 2 Faculty of Chemistry, Biological and Chemical Research Centre, University of Warsaw, 02-089 Warsaw, Poland; 3 Faculty of Science and Engineering, Johan Gadolin Process Chemistry Centre, Centre for Process Analytical Chemistry and Sensor Technology (ProSens), Åbo Akademi University, 20500 Turku, Finland; 4 Department of Biophysics, Warsaw University of Life Sciences—SGGW, 02-776 Warsaw, Poland; krzysztof_dolowy@sggw.pl

Ion sensors, conventionally known as ion-selective membrane electrodes, were devised 100 years ago with the invention of a pH electrode with a glass membrane (in 1906 Cremer, in 1909 Haber and Klemensiewicz). The electrode type had a symmetric design with an internal contact made with a solution. Interestingly, in the same period, membrane biophysics made a great step forward. It was hypothesized that the nerve cell is covered by an invisible membrane specifically selective to potassium ions. Since the concentration of potassium ions in the cytoplasm is high, and in the external medium low, the membrane potential is negative and during nerve impulse it nullifies (Bernstein, 1912).

Today, in ion-sensor technology, there are many membranes made of glass, solid-state, plastics, and composites, as well numerous internal contacts made of conducting polymers, carbon nanotubes, graphene, conducting clays, and composites. Owing to these advances, sensors can be miniaturized, made service-free, and produced by a mass fabrication technique, such as 3D printing. The sensor responses are now interpreted by models that are able to access the time and space domains. In this way, sensor signals may be mathematically transposed. Supported by advanced modeling, ion sensors are now deliberately calibration-free, and quality checking can be automated. The sensors can be used in ad hoc and routine application areas like clinical care and diagnostics, monitoring personal physiological performance in sport, and measuring water quality.

The heart of the ion sensors is always the same: a membrane that can develop the ion response due to selective, fast, and reversible ion exchange at the sample–membrane interface. The membrane can be artificial or biological.

This Special Issue presents major progress that has been made recently in ion-sensor technology. Scientific activity in this field is currently very intense and is characterized by several hundred published papers. The editors have by no means aimed to cover exhaustively all subjects. We, the editors, briefly elucidate the background to the recent trends in order to introduce the reader to the more detailed reports by different authors with references, which are gathered here in this Special Issue volume.

Conventional membrane ion-selective electrodes (ISEs) contain the ion-sensitive membrane that separates the internal contact (with connecting wire to the electrometer) and solution (containing the analyte of interest). From the very beginning, the electrodes with a solid membrane containing insoluble inorganic salts were equipped with a solid contact (metal wire, e.g., Ag or Pt, carbon rod) while the ISEs with liquid and derived plastic membranes containing ion exchangers or neutral ligands were equipped with a so-called internal solution and internal reference electrode, usually Ag, AgCl electrode, immersed in the solution. Interestingly, historically the first ISE pH electrode employed a glass membrane which is an overcooled liquid in solid-state at room temperature. The internal solution typical for this popular electrode ignited a search for a solid contact by the Nikolskii group, known for their pioneering contributions dedicated to the glass electrode. The idea was to find a material that could couple hydrogen ion sensitivity. For this purpose, alkaline metal alloys (e.g., Li or Na in Sn) were used to coat the inner side of the glass membrane used [1].

The elimination of the internal solution in ion-selective membrane electrodes and substituting with plastic membranes (usually poly (vinyl chloride), PVC) and in reference electrodes has proved by far to be challenging. This approach aroused intense research interest, which has lasted up to today. No wonder, since the ISE plastic membranes are known for their sensitivity to ions of physiological importance, like sodium, potassium, calcium, magnesium, chlorides, and bicarbonates, which are extensively measured in medical theatres. The need for miniaturization of these ISEs while keeping them robust, durable, cheap, and maintenance-free accompanied the expectation of automated clinical measurement. The answer to this challenge was offered by substituting the liquid contact with a solid contact made from a conducting polymer (CP) layer, as reported by Lewenstam and co-workers in 1992 [2]. The first solid-contact sodium-selective electrode with a plastic membrane was described, thus opening a new chapter in the electrochemical analysis of ions, especially blood electrolytes. Conducting polymer is a versatile material that addresses several demands in ion-sensor technology. CP is a solid phase and does not evaporate. CP is characterized by mixed conductivity, intrinsic ion-to-electron coupling, it is a material with free electrons mediating at the CP-lead to meter interface and offers the possibility of intentional ligand doping which allows tailoring the desired ionic sensitivity by ion exchange with bathing solution. Additionally, CPs can be soluble and processable.

On its own, a CP membrane can be an electrode membrane for anions, cations, or electrons (redox electrode) [3,4] or an ionically insensitive CP-based membrane reference electrode [5]. CP can also play a role of ion-to-electron transducer, when covered with an ISE plastic membrane [6,7]. In recent years, the concept of a solid contact is reinforced by use of nanostructures deposited in thin layers of, e.g., carbon in different 3D forms and of carbon doped by, or directly used, nanostructures of metals, transition (redox) metal oxides, salts and ligands with redox groups, or carbon nanostructures with CPs. In this way the electrochemical charge coupling could be downscaled to nano. The materials, their benefits and limits are known from supercapacitors. Developing an electrochemically accessible surface area and downscaling to nano dimensions increase the interfacial capacitance of the solid contact, and the apparent stability of the contact is observed [8,9]. A new wave that just enhanced solid contact technology is represented by the use of 3D printable composite PVC-based materials as the electrochemical membranes of all-solid-state chemical ion-sensors and reference electrodes [10]. Since PVC ISE membranes and reference composites contain hidden water, the sandwich of these two elements forms a “well-wetted” solid contact [11].

As mentioned, the papers collected here reflect the main trends and challenges, actual applications and potential.

In the very empirical report on plastic membranes with dispersed equitransferent electrolytes, Lingenfelter and co-authors [12] showed how to obtain the reference electrodes. The research in this area is governed by the idea that there is a wide insensitivity towards ions in the matrices, which is opposite to the aim of ion sensors. Since the reference electrode in experimental applications typically serves several indicator ISEs, the research in ISEs is distinctively more laborious in comparison to indicator electrodes. The report shows for the first time in detail a comprehensive, semi-industrial multi-solution protocol on testing the reference electrodes and the criteria for selecting the most suitable one.

There are three papers that represent crucial pre-set topics in ion sensors.

Maksymiuk et al. [13] review the unfavorable effects of dissolution of the solid-contact material in the plastic membrane. In a convincing way, the authors underline a possible mutual influence of CPs on the properties of plastic ISE membrane and vice-versa. Lenar et al. [14] describe an application of ruthenium oxide as the mediating material in a solid-contact potassium-sensitive ISE. The authors point out the increase of redox capacity and response reproducibility when the hydrated ruthenium oxide is used.

There is an intriguing link between these two papers which directly inspires a new research pathway. For a long time, there has been the belief that an ideal solid contact should not allow a water layer formation between the solid contact and the ISE membrane (even if it is known that PVC ISE membranes contain “hidden water”). A strong theoretical argument is supported by so-called water tests which show the sluggish response if the water layer is present at the contact–membrane interface. Maksymiuk et al. [13] correctly argue though that the conventional electrodes with internal solution are by far more stable: “a classic ISM-containing arrangement offers high stability of potential readings in time due to well-defined, reversible ion-electron transfer through all sensor interfaces”. On the other hand, in the paper of Lenar et al. [14], we can read that “the occurrence of structural water in RuO_2_•xH_2_O, which creates a large inner surface available for ion transport and was shown to be a favourable feature in the context of designing potentiometric sensors”. Accordingly, the authors provide evidence that the solid contact with chemically bounded hydration water in it can be as good as highly hydrophobic. The former case does not allow for the water layer formation, and the latter has sufficient water content to stand water transport through the membrane decreased by a small gradient of chemical potential for water between the “well-wetted” solid contact and ISE membrane (the same argument can be applied for ion transmembrane fluxes and mechanical membrane–contact adhesion making a water test redundant). In this situation, as correctly commented by Maksymiuk et al. [13] an optimization of the sensor with solid contact depends on a lot of factors and in the last instance the experimental theatre in which the ion-sensors are used.

Lindner et al. [15] present the application of ion-sensitive membranes in nonzero-current measurement. The authors convincingly demonstrate the utility of the plasticized PVC membrane modified working electrode for the voltammetry measurement of highly lipophilic molecules. Nonzero-current measurement has an additional value vs. the zero current mode described above, since the ISE response can be described without ambiguity by equivalent circuits. In-zero current, i.e., potentiometric ISEs, the equivalent circuit of a pseudocapacitor, implying faradaic processes at solid contact–membrane interface is predominantly used. However, recently, the more rigorous double-layer capacitor, with its simple equivalent circuit reflecting charge interactions only, is as well considered.

Finally, in the review by Lyu et al. [16] the responses of solid-contact electrodes are highlighted by listing the various materials used and providing a very basic insight into theoretical principles. The authors dedicate their preview to a hot issue of present sensor technology, namely wearable sensors (see below).

Wearable solid-contact sensors, their fabrication, challenges, and perspectives are reviewed by Lyu et al. [16]. The authors show several formulations that can serve in the screening of electrolytes in sweat. The development and market are highly anticipated due to growing popularity of sports like the marathon or triathlon. Monitoring body dehydration or electrolyte imbalance plays a crucial role in the well-being and success of participants.

ISEs are often used for automated determination of ion concentration in whole blood or plasma. In such measurements, the electrode is usually washed and calibrated before each measurement within a few seconds. Zajac et al. [17] show that the use of ISEs might be a reasonable choice to study ion transport across epithelial cells. However, the number of ions transported is very small. The only possibility to measure the change in ion concentration by an ISE in the solution facing the living cells is to drastically reduce its volume [18]. In addition, the washing and calibration procedure for an ISE facing living cells must be changed. The ISE must be calibrated before the experiment and when mounted on the measuring platform the one-point calibration in the cell’s friendly medium can be performed. The ISE used must be miniaturized and be able to survive for an hour of measurements without its properties changing. The use of sodium, potassium, pH, and chloride electrodes provided insight into the mechanism of ion and water transport across the epithelium.

The Jasielec et al. [19] paper is a theoretical one that describes the transport of ions into living mitochondria. Since mitochondria have a high negative inner potential caused by expulsing proton ions at the expense of metabolic energy, they are prone to inward transport of cations into the matrix. The transport of cations into the matrix occurs via ion channels present in the mitochondrial membrane, which are precisely controlled by physiology. Mitochondria are especially effective in transporting calcium ions into the matrix. Since at the same time phosphates are also transported, calcium phosphate gel is formed in the matrix. The paper of Jasielec et al. [19] analyze the role that magnesium, carbonate, and hydroxyl ions play in the process of formation of osmotically inactive gel in the matrix formed in the multi-ion complex environment.

It would be rewarding to penetrate a mitochondrial membrane to measure free magnesium and calcium in the matrix. This calls for nanoelectrodes. Are they beyond limits?

Without a doubt, the electroanalytical application of membranes has come to a new period which is marked by the advent of the anisotropic heterogenous composite membrane. Therefore, this breakthrough brings with it the need for future research in 3D modeling, 3D print-fabrication, multiscale applications, and associated multidimensional challenges.
